# Postoperative complications after stapled and hand‐sewn ileal pouch‐anal anastomosis for familial adenomatous polyposis: A multicenter study

**DOI:** 10.1002/ags3.12019

**Published:** 2017-07-20

**Authors:** Tsuyoshi Konishi, Hideyuki Ishida, Hideki Ueno, Hirotoshi Kobayashi, Takao Hinoi, Yasuhiro Inoue, Fumio Ishida, Yukihide Kanemitsu, Tatsuro Yamaguchi, Naohiro Tomita, Nagahide Matsubara, Toshiaki Watanabe, Kenichi Sugihara

**Affiliations:** ^1^ Department of Gastroenterological Surgery Gastroenterological Center Cancer Institute Hospital of the Japanese Foundation for Cancer Research Tokyo Japan; ^2^ Study Group for Familial Adenomatous Polyposis in the Japanese Society for Cancer of the Colon and Rectum Tokyo Japan; ^3^ Department of Digestive Tract and General Surgery Saitama Medical Center Saitama Medical University Saitama Japan; ^4^ Department of Surgery National Defense Medical College Saitama Japan; ^5^ Department of Gastrointestinal Surgery Tokyo Medical and Dental University Tokyo Japan; ^6^ Department of Gastroenterological and Transplant Surgery Hiroshima University Hiroshima Japan; ^7^ Department of Gastrointestinal and Pediatric Surgery Mie University Graduate School of Medicine Mie Japan; ^8^ Digestive Disease Center Northern Yokohama Hospital Showa University Yokohama Japan; ^9^ Colorectal Surgery Division National Cancer Center Hospital Tokyo Japan; ^10^ Department of Surgery Tokyo Metropolitan Cancer and Infectious Diseases Center Komagome Hospital Tokyo Japan; ^11^ Division of Lower GI Surgery Department of Surgery Hyogo College of Medicine Hyogo Japan; ^12^ Department of Surgical Oncology Graduate School of Medicine The University of Tokyo Tokyo Japan; ^13^ Tokyo Medical and Dental University Tokyo Japan

**Keywords:** familial adenomatous polyposis, ileal pouch‐anal anastomosis, total proctocolectomy

## Abstract

Ileal pouch‐anal anastomosis (IPAA) after total proctocolectomy (TPC) can be conducted with either hand‐sewn or stapled anastomosis for patients with familial adenomatous polyposis (FAP). Although stapled IPAA without mucosectomy has a higher risk for developing adenomas in the remnant mucosa, it is the simpler procedure with potential benefit in short‐term outcomes. However, it remains controversial as to whether stapled IPAA has any advantages in reducing postoperative complications. The aim of the present study was to compare the postoperative complications and short‐term outcomes of stapled and hand‐sewn IPAA for patients with FAP, using a multicenter cohort sample in Japan. Data of 143 patients with FAP who underwent TPC with stapled IPAA (n=37) and hand‐sewn IPAA (n=106) at 23 institutions between 2000 and 2012 were collected. Postoperative complications, proportion of ostomy, fecal continence and overall survival were compared. Overall rates of the Clavien‐Dindo grade II‐IV complications were not different between the two groups (19% in stapled vs 25% in hand‐sewn, *P*=.42), with significantly fewer pouch‐related complications including leakage, pelvic abscess, vaginal fistula and anastomotic stricture in stapled IPAA (none in stapled vs 11% in hand‐sewn, *P*=.036). There was no mortality. Proportion of ostomy at 12 months was similar (2.7% in stapled vs 4.3% in hand‐sewn, *P*=.26). Mean Wexner score was similar. (0.47 in stapled vs 2.0 in hand‐sewn, *P*=.12). Five‐year overall survival excluding Stage IV patients was 96% in both groups. Stapled IPAA is a safe option in patients with FAP with a potential benefit in reducing pouch‐related complications.

## 1 INTRODUCTION

Familial adenomatous polyposis (FAP) is an autosomal dominant disease characterized by the development of numerous colorectal adenomatous polyps which can lead to cancer often by the fourth decade.^1^ Total proctocolectomy (TPC) with ileal pouch‐anal anastomosis (IPAA) has been established as a standard procedure for minimizing the risk of cancer death. Since the first report by Parks and Nicholls in 1978,^2^ IPAA was originally conducted by hand‐sewn technique with mucosectomy down to the dentate line to eliminate all mucosa at risk. With the development of staple devices in the 1980s, stapled IPAA without mucosectomy has been increasingly conducted.^3^, ^4^, ^5^, ^6^ Previous studies have reported several pros and cons on these two anastomotic techniques. Stapled IPAA is the simpler type of anastomosis with shorter length of operation and better fecal continence possibly as a result of omission of mucosectomy and preservation of the anal transitional zone (ATZ).^5^, ^7^, ^8^, ^9^, ^10^ In contrast, hand‐sewn IPAA with mucosectomy can reduce a substantial risk for developing adenomas at the anastomotic site.^11^

Previous studies have shown inconsistent results on postoperative complications after these two procedures. Some studies found higher incidence of septic complications, fistula and anastomotic stricture after hand‐sewn IPAA,^5^, ^9^, ^12^ whereas other studies showed a trend toward higher incidence of overall complications and stricture after stapled IPAA.^13^, ^14^ Importantly, most of the previous studies included patients with ulcerative colitis (UC) rather than patients with FAP 5, 8, 14, 15 although the complication rates were different between such patients.^5^, ^8^, ^14^, ^15^ Furthermore, none of the studies used objective criteria such as the Clavien‐Dindo classification for stratifying the complications.^5^, ^8^, ^9^, ^12^, ^13^, ^14^, ^15^ These limitations could explain inconsistent results in the previous studies, and we need evidence that focuses on patients with FAP with the use of objective criteria for assessing complications. The aim of the present study was to compare postoperative complications and short‐term outcomes after stapled and hand‐sewn IPAA for patients with FAP using multicenter data in Japan.^16^, ^17^, ^18^

## 2 MATERIALS AND METHODS

### 2.1 Original data sources for this study

Original data for this study were compiled from 23 institutions that are members of the Japanese Society for Cancer of the Colon and Rectum (JSCCR), which includes the departments of surgery, internal medicine, pathology, and radiology at hospitals throughout Japan.^19^, ^20^ All patients diagnosed as having FAP and undergoing colorectal resection in each institution between the years 2000 and 2012 were retrospectively collected and registered for the database as described previously.^16^, ^17^, ^18^ Patients having a previous history of colorectal resections were excluded from the database to avoid double registration. The diagnosis of FAP was established if patients met any of the following three criteria according to the 2012 JSCCR Clinical Practice Guidelines for Hereditary Colorectal Cancer:^21^, ^22^ (i) patients with 100 or more adenomatous polyps in the colon with or without a family history of FAP; (ii) patients with fewer than 100 adenomatous polyps in the colon with a family history of FAP; and (iii) patients with germline mutations in the adenomatous polyposis coli (*APC*) gene. We defined FAP patients with ≥1001 polyps in the colon and rectum as having profuse phenotype, 100‐1000 polyps as sparse phenotype, and ≤99 polyps as attenuated phenotype. This was a retrospective observational cohort study and was approved by the ethical committees of the JSCCR and an institutional reviewer board of each participating institution.

### 2.2 Patient selection and data extraction

Data of all patients undergoing TPC with IPAA were extracted from the database. Clinical variables, postoperative complications and overall survival were compared between patients undergoing stapled IPAA and hand‐sewn IPAA.

### 2.3 Primary and secondary endpoints

Primary endpoint of the present study was the rate of postoperative complications which were stratified according to the Clavien‐Dindo classification.^23^ Evaluated complications included anastomotic leakage, pelvic abscess, vaginal fistula, anastomotic stricture, ileus/bowel obstruction, wound infection, cardiovascular event and others. Pouch‐related complications were defined as described previously by Ganschow et al.,^13^ including anastomotic leakage, pelvic abscess, vaginal fistula and anastomotic stricture. Extra‐pelvic complications were defined as those other than pouch‐related complications. Secondary endpoints included proportion of ostomy after surgery, anal function evaluated by the Wexner fecal incontinence score, overall survival after surgery and incidence of desmoid tumors. The Wexner fecal incontinence score consisted of the score sum of five parameters (frequency of gas, liquid or solid incontinence, need to wear a pad and lifestyle alterations) scored on a scale of 0 (absent) to 4 (daily).^24^ A total score of 0 suggested full continence and a score of 20 complete fecal incontinence. Data on the Wexner fecal incontinence score were collected retrospectively from medical charts at the time of registration to the study.

### 2.4 Statistical analysis

Statistical analysis was carried out using JMP software V 9.0.0 (SAS Institute, Cary, NC, USA). To compare stapled and hand‐sewn IPAA, univariate analysis was done using Pearson's χ^2^‐test or Fisher's exact probability test for categorical variables and Wilcoxon/Kruskal‐Wallis rank‐sum test for continuous variables. Survival, incidence of desmoid tumors and proportion of ostomy after surgery were analyzed using the Kaplan‐Meier method and log‐rank test. Patients who were alive and with ostomy at the last follow up were treated as censored, respectively. *P* values <.05 were considered to be significant.

## 3 RESULTS

A total of 303 patients with FAP undergoing colorectal resection were registered in the database, and 190 of them underwent restorative TPC. Median number of patients with restorative TPC registered to the database per the center was six (range: 1‐65). After excluding procedures without pouch creation (n=13), without information on reconstruction (n=13) and without information on the Clavien‐Dindo Classification of postoperative complications (n=21), a total of 143 patients undergoing TPC with stapled or hand‐sewn IPAA were eligible for the study. The cohort included 37 patients (26%) undergoing stapled IPAA and 106 patients undergoing hand‐sewn IPAA (74%). Proportion of stapled IPAA was 30% (20/66) in 2000‐2006 and 22% (17/77) in 2007‐2012, showing no significant change during the study period (*P*=.26). Proportion of stapled IPAA was not statistically different between the centers with lower volume (median or less) and higher volume (10/26, 38% vs 27/117, 23%, *P*=.11)

### 3.1 Patient characteristics and surgical background

Patient characteristics and surgical procedures are shown in Table 1. There were no differences regarding patient characteristics or surgical background between the two groups. Median age was 30‐31 years, and gender was equally distributed. Majority of the patients had the profuse or sparse phenotype, and nearly half of the indications for surgery were cancer in both groups. Proportion of laparoscopic surgery was 59% and 45% in stapled and hand‐sewn IPAA, respectively. Covering ileostomy was created in 59% and 65% in stapled and hand‐sewn IPAA, respectively. Operation time and bleeding volume were not significantly different between the groups. Median follow‐up period was 41 months after stapled IPAA and 52 months after hand‐sewn IPAA (*P*=.84).

**Table 1:** Characteristics and surgical backgrounds of 143 patients with FAP who underwent TPC with stapled IPAA and hand‐sewn IPAA

	Stapled IPAA (n=37)	Hand‐sewn IPAA (n=106)	*P* value
Gender (male)	19 (51%)	53 (50%)	0.89
Median age, years (IQR)	31 (21‐39)	30 (24‐37)	0.94
Phenotype			0.32
Profuse (polyps ≥1001)	11 (30%)	44 (42%)	
Sparse (polyps 100‐1000)	22 (59%)	56 (53%)	
Attenuated (polyps ≤99)	4 (11%)	6 (5.7%)	
Indication for surgery			0.07
Prevention	17 (46%)	54 (51%)	
Symptom	3 (8.1%)	1 (0.9%)	
Cancer	17 (46%)	51 (48%)	
Stage 0‐I	6 (16%)	26 (25%)	
Stage II	1 (2.7%)	9 (8.5%)	
Stage III	5 (14%)	14 (13%)	
Stage IV	3 (8.1%)	1 (0.9%)	
Unknown M0	2 (5.4%)	1 (0.9%)	
Approach (laparoscopic)	22 (59%)	48 (45%)	0.14
Covering ileostomy (present)	22 (59%)	69 (65%)	0.54
Median operation time, min (IQR)	376 (289‐478)	353 (272‐538)	0.91
Median bleeding, mL (IQR)	250 (65‐425)	297 (150‐485)	0.093
Median follow up, months (IQR)	41 (20‐89)	52 (26‐78)	0.84

FAP, familial adenomatous polyposis; IPAA, ileal pouch‐anal anastomosis; TPC, total proctocolectomy.

### 3.2 Postoperative complications

Table 2 shows the details of postoperative complications according to the Clavien‐Dindo classification. There was no mortality among the study population. Overall rates of grade II‐IV complications were 19% in stapled IPAA and 25% in hand‐sewn IPAA, which were not significantly different between the two groups (*P*=.42). Overall rates of grade III‐IV severe complications were 8.1% in stapled IPAA and 19% in hand‐sewn IPAA. Although showing a clear trend, the difference did not reach statistical significance (*P*=.13). However, stapled IPAA was associated with significantly fewer grade II‐IV pouch‐related complications than hand‐sewn IPAA (none vs 11%, *P*=.036). Overall rates of grade III‐IV severe pouch‐related complications were also marginally fewer in stapled IPAA (none vs 9.4%, *P*=.064). Anastomotic stricture was the most frequent pouch‐related complication followed by pelvic abscess after hand‐sewn IPAA. In contrast, overall rates of extra‐pelvic complications were not different between the groups. Ileus/bowel obstruction was the most frequent complication in both groups.

**Table 2:** Postoperative complications according to the Clavien‐Dindo classification in patients with FAP who underwent TPC with stapled IPAA and hand‐sewn IPAA

	Complications ≥ Grade II			Complications ≥ Grade III		
	Stapled IPAA (n=37) n (%)	Hand‐sewn IPAA (n=106) n (%)	*P* value	Stapled IPAA (n=37) n (%)	Hand‐sewn IPAA (n=106) n (%)	*P* value
Overall complications	7 (19)	27 (25)	.42	3 (8.1)	20 (19)	.13
Pouch‐related complications						
Overall pouch‐related	0 (0)	12 (11)	.036	0 (0)	10 (9.4)	.064
Anastomotic leakage	0 (0)	2 (1.9)	1.00	0 (0)	1 (0.9)	1.00
Pelvic abscess	0 (0)	5 (4.7)	.33	0 (0)	4 (3.8)	.57
Vaginal fistula^a^	0 (0)	1 (1.9)	1.00	0 (0)	1 (1.9)	1.00
Anastomotic stricture	0 (0)	6 (5.7)	.34	0 (0)	6 (5.7)	.34
Extra‐pelvic complications	7 (19)	16 (15)	.59	3 (8.1)	11 (10)	.69
Ileus/bowel obstruction	7 (19)	11 (10)	.18	3 (8.1)	6 (5.7)	.70
Wound infection	0 (0)	4 (3.8)	.57	0 (0)	4 (3.8)	.57
Cardiovascular event	0 (0)	1 (0.9)	1.00	0 (0)	1 (0.9)	1.00
Other^b^	0 (0)	1 (0.9)	1.00	0 (0)	0 (0)	–

a Analysis was carried out in female patients.

b Anemia requiring transfusion.

FAP, familial adenomatous polyposis; IPAA, ileal pouch‐anal anastomosis; TPC, total proctocolectomy.

### 3.3 Proportion of ostomy and anal function

**Figure 1:**
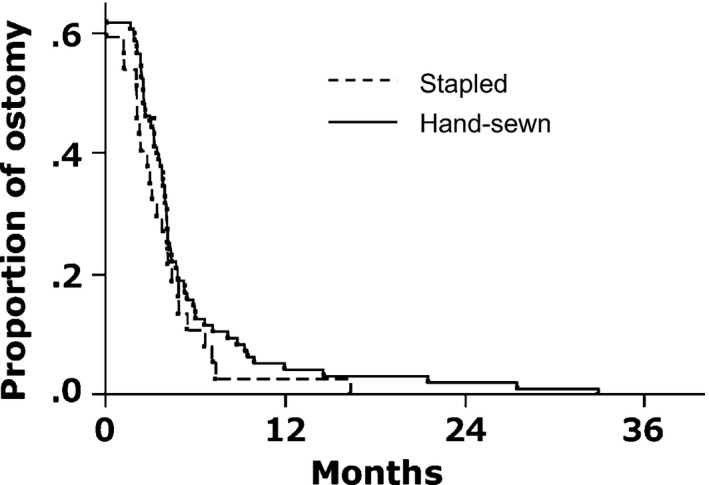
Proportion of ostomy after ileal pouch‐anal anastomosis (IPAA). There was no difference in proportion of ostomy after stapled and hand‐sewn IPAA. Proportion of ostomy was 11% and 13% at 6 months, and 2.7% and 4.3% at 12 months in stapled and hand‐sewn IPAA, respectively (*P*=.26)

Figure 1 shows the proportion of ostomy after surgery. The proportion of ostomy was similarly decreased in the two groups (11% and 13% at 6 months, 2.7% and 4.3% at 12 months in stapled and hand‐sewn IPAA, respectively). Median duration from IPAA to ostomy closure was 108 days after stapled IPAA and 120 days after hand‐sewn IPAA (Table 3). After hand‐sewn IPAA, two patients had ileostomy at the last follow up as a result of poor anal function (n=1) and persistent vaginal fistula (n=1). Mean Wexner fecal continence score showed that both groups achieved adequate anal function after ostomy closure (0.47±0.84 vs 2.0±0.45, *P*=.12).

**Table 3:** Ostomy and fecal incontinence score in patients with FAP who underwent TPC with stapled IPAA and hand‐sewn IPAA

	Stapled IPAA (n=37)	Hand‐sewn IPAA (n=106)	*P* value
Ostomy present at the last follow up	0 (0%)	2 (2.0%)^b^	1.00
Median days before ostomy closure (IQR)^a^	108 (67‐152)	120 (80‐160)	.36
Mean Wexner score^b^ (SD)	0.47 (0.84)	2.0 (0.45)	.12

Analysis was carried out excluding unknown cases (n=5).

a Analysis was carried out in patients with covering ileostomy.

b Analysis was carried out in patients without ostomy at the last follow up.

FAP, familial adenomatous polyposis; IPAA, ileal pouch‐anal anastomosis; IQR, interquartile range; SD, standard deviation; TPC, total proctocolectomy.

### 3.4 Overall survival and incidence of desmoid tumors

**Figure 2:**
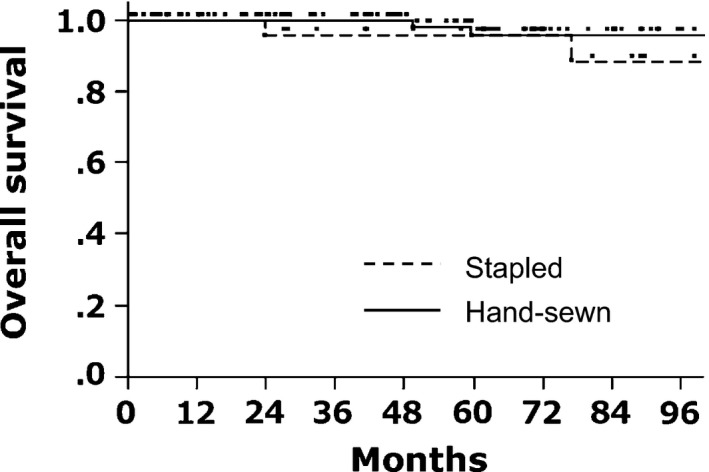
Overall survival after ileal pouch‐anal anastomosis (IPAA). There was no difference in overall survival after stapled and hand‐sewn IPAA. Patients with Stage IV cancer were excluded from the analysis. Five‐year overall survival was 96% in the two groups (*P*=.15)

Figure 2 shows overall survival after surgery excluding patients with Stage IV cancer. Overall survival was not significantly different between the two groups, showing 96% survival at 5 years in both groups. The overall survival was also similar between the groups for the entire population including Stage IV disease (90% vs 94% at 5 years in stapled and hand‐sewn IPAA, respectively, *P*=.13), after excluding the patients with cancer (94% vs 100% at 5 years in stapled and hand‐sewn IPAA, respectively, *P*=.11) or comparing cancer‐associated patients without Stage IV disease (100% vs 92% at 5 years in stapled and hand‐sewn IPAA, respectively, *P*=.51). There was no difference in incidence of desmoid tumors between the two anastomotic procedures (18% vs 23% at 5 years in stapled and hand‐sewn IPAA, respectively, *P*=.5937).

## 4 DISCUSSION

The present multicenter retrospective study analyzed short‐term outcomes of a total of 143 patients who underwent restorative TPC for FAP in Japan, including 37 stapled IPAA and 106 hand‐sewn IPAA. Hand‐sewn IPAA with mucosectomy is generally accepted as a time‐consuming and complicated procedure.^25^ Hence, in Western countries, this technique has been increasingly replaced by stapled IPAA without mucosectomy.^3^, ^4^, ^5^, ^6^ In a recent case‐matched study from Germany which focused on FAP patients,^13^ anastomotic technique was switched from hand‐sewn IPAA to stapled IPAA in 2001, and stapled IPAA during 2001‐2006 was compared with hand‐sewn IPAA during 1984‐2001. The authors reported 31% and 23% pouch‐related complications in stapled and hand‐sewn IPAA, respectively. In a consecutive series of 119 restorative TPC for FAP during the period from 1982 to 1999 by Remzi et al.,^9^ stapled IPAA was introduced from 1988 and finally accounted for 65% of the study population. In Japan, the present data clearly showed that surgeons consistently preferred hand‐sewn IPAA during the study period of 2001‐2012, and stapled IPAA has been conducted rather selectively, although patient characteristics and surgical backgrounds were similar between the groups. Regarding IPAA, the data revealed excellent short‐term outcomes in both anastomotic techniques. Although direct comparison of the complication rates between the studies might be difficult because of different definitions of the complications, the rates in the present study seemed somewhat favorable compared with previously published results for FAP patients.^13^, ^26^, ^27^ This could be partially explained by the newer study period of the present series in which surgical devices and techniques were much improved compared with the period before 2000. In addition, a high proportion of covering ileostomy of over 60% could have contributed to reduce anastomotic complications.

There have been conflicting results on the advantages of stapled IPAA in reducing postoperative complications over hand‐sewn IPAA. Although some authors showed favorable results toward stapled IPAA,^5^, ^9^, ^12^ others reported no benefit or a trend toward higher complications.^13^, ^14^ In the present study, the analysis revealed no significant difference in the rates of overall complications or extra‐pelvic complications between stapled and hand‐sewn IPAA. However, the data showed significantly fewer grade II‐IV pouch‐related complications and marginally fewer grade III‐IV severe pouch‐related complications in stapled IPAA. Although stapled IPAA was rather selectively carried out in this series, patient characteristics and surgical backgrounds were not different between the groups, including proportion of laparoscopic surgery or covering ileostomy. The data indicated at least equivalent safety of stapled IPAA compared with hand‐sewn IPAA, with a potential benefit in reducing pouch‐related complications in patients with FAP.

Previous studies reported better fecal continence after stapled IPAA without mucosectomy compared with hand‐sewn IPAA with mucosectomy, possibly as a result of reduced anal canal manipulation and preservation of ATZ.^5^, ^7^, ^8^, ^9^, ^10^ In contrast, the studies by Remzi et al. and Ganschow et al. reported no difference in function between these two techniques.^9^, ^13^ In the present study, ostomy was successfully closed except in two patients after hand‐sewn IPAA, and the patients in both groups achieved adequate anal function. A previous article reported worse functional outcomes after pelvic sepsis in patients who underwent IPAA.^28^ The low pouch‐related complication rate in the present study could have contributed to the excellent functional outcomes.

Another important discussion on stapled IPAA is a substantial risk of developing adenomas and cancers at the anastomotic site as a result of remnant mucosa. Indeed, previous studies reported a reduced incidence of adenomas after hand‐sewn IPAA compared with stapled IPAA.^11^, ^29^ On this point, choice of anastomotic procedure should not be based simply on the complication rates, and patients with FAP who receive stapled anastomosis should be fully informed about the high risk of developing adenoma. However, it should be noted that the risk of developing adenomas is not totally eliminated even after hand‐sewn IPAA with mucosectomy. Von Roon et al.^29^reported a cumulative risk of developing adenoma of 22.6% at 10 years, and van Duijvendijk et al.^11^reported such risk as 10% at 7 years after hand‐sewn IPAA with mucosectomy. Furthermore, another study revealed that development of cancer was not different between the two anastomotic techniques.^30^ In light of these findings, all patients with FAP should undergo endoscopic surveillance at regular intervals regardless of the anastomotic procedure. Unfortunately, in the present study, data on postoperative development of adenomas and cancers were not available. Although 5‐year overall survival was similar between the two groups, overall survival did not reflect the risk of recurrent adenoma or cancers. Future study with a longer follow‐up period is needed to compare the risk for recurrent adenomas or cancers.

There are several limitations in the present study. The study remains retrospective in nature and has the limitations inherent to this type of design. Data were based on retrospective review of the chart and, accordingly, diagnostic criteria of complications or follow‐up method could have varied among the centers or surgeons. Data on the detailed procedure of hand‐sewn or stapled IPAA, or how stapled IPAA was selected were not available. Although it would be ideal to have prospective studies to address these points, there have been few prospective trials on this disease because of its rare incidence and the small number of available cases. A comparison was made without a case‐matched design, but the patient characteristics and surgical backgrounds were adequately equivalent for comparison of the two groups. Although this was a nationwide multicenter study including 143 patients with FAP, the number of stapled IPAA was relatively small and not powered to draw definite conclusions on the outcomes. A larger multicenter trial or meta‐analysis is needed to address this concern. Small numbers of cases per institution were another limitation of the present study. Data related to surgeon's skill on IPAA were not available, including number of involved surgeons or the experience of IPAA per surgeon. However, we believe that quality of clinical practice in this dataset represented the results of leading academic institutions or cancer centers in Japan, as all of the 23 institutions were members of the JSCCR and met the criteria to take a leading role in the treatment of colorectal cancer in Japan. Some important parameters on FAP patients were not available in the database, including proband or call‐up cases. Finally, 34 cases (18%) were excluded from the analysis as a result of missing data on pouch reconstruction or postoperative complications, which could have caused a bias. Despite these limitations, we believe that this study reflects the actual outcomes of stapled and hand‐sewn IPAA in this study setting.

In conclusion, grade II‐IV pouch‐related complications were fewer in stapled IPAA compared to hand‐sewn IPAA in patients with FAP, whereas there were no differences in incidence of overall complications, fecal incontinence score, proportion of ostomy and overall survival between the two procedures. Stapled IPAA is a safe option in patients with FAP with a potential benefit in reducing pouch‐related complications.

## DISCLOSURE

Conflict of Interest: Drs Tsuyoshi Konishi, Hideyuki Ishida, Hideki Ueno, Hirotoshi Kobayashi, Takao Hinoi, Yasuhiro Inoue, Fumio Ishida, Yukihide Kanemitsu, Tatsuro Yamaguchi, Naohiro Tomita, Nagahide Matsubara and Kenichi Sugihara have no conflicts of interest or financial ties to disclose. Dr Toshiaki Watanabe has received lecture fees from Ethicon, Covidien and Olympus. This study was supported in part by a Grant‐in‐Aid for Cancer Research from the Ministry of Health, Labour and Welfare, by the Japanese Society for Cancer of the Colon and Rectum, and by the JFE Grant. The funding source had no role in the design, practice or analysis of this study.
